# Combination of *Astragalus membranaceus* and *Panax notoginseng* as Main Components in the Treatment of Diabetic Nephropathy: A Systematic Review and Meta-Analysis

**DOI:** 10.1155/2023/2945234

**Published:** 2023-04-17

**Authors:** Xiaoning An, Youhua Xu, Dingkun Gui

**Affiliations:** ^1^Department of Nephrology, Shanghai Sixth People's Hospital Affiliated to Shanghai Jiao Tong University School of Medicine, Shanghai, China; ^2^Faculty of Chinese Medicine, State Key Laboratory of Quality Research in Chinese Medicine, Macau University of Science and Technology, Macao, China

## Abstract

**Objective:**

This meta-analysis evaluated the curative effect of the compatibility of *Astragalus membranaceus* and *Panax notoginseng* (ARPN) as main components on diabetic nephropathy.

**Methods:**

We used various Chinese and English databases, including the Cochrane Library, PubMed, Embase, Web of Science, the China National Knowledge Infrastructure (CNKI), China Biology Medicine Disc (SinoMed), VIP, and Wanfang, to search for randomized controlled trials on the compatibility of *Astragalus membranaceus* and *Panax notoginseng* as main components. After data extraction, meta-analysis was performed with Review Manager 5.4.0 and Stata 15, and the Grading of Recommendations Assessment, Development, and Evaluation (GRADE) framework was used to evaluate the quality of the evidence.

**Result:**

A total of 17 studies involving 1342 patients with diabetic nephropathy were included. Compared with the control group, ARPN can significantly improve the clinical effective rate of diabetic nephropathy (OR 5.12, 95% CI 3.42 to 7.66, *P* < 0.00001), and the curative effect of reducing UAER (MD −26.67, 95% CI −31.30 to −22.04, *P* < 0.00001) and 24 h urinary protein (SMD −0.58, 95% CI −0.75 to −0.41, *P* < 0.00001) is also significantly better than that of the control group, and it can also improve the renal function(Scr: MD −13.78, 95% CI −25.39 to −2.17, *P*=0.02; BUN: MD −0.74, 95% CI −1.27 to −0.20, *P*=0.007). In addition, it can also reduce glycosylated hemoglobin (SMD −1.30, 95% CI −2.33 to −0.27, *P*=0.01) and blood lipid(TC: SMD −0.62, 95% CI −0.95 to −0.29, *P*=0.0002; TG: SMD −0.47, 95% CI −0.75 to −0.19, *P*=0.0009; LDL: SMD −0.43, 95% CI −0.68 to −0.18, *P*=0.0008), and improve the TCM syndrome score (MD −4.87, 95% CI −6.17 to −3.57, *P* < 0.00001). Subgroup analysis suggested that the treatment plan of the control group could be the sources of heterogeneity. All the included studies had no obvious adverse effects.

**Conclusions:**

The compatibility of Radix Astragali and Radix notoginseng as the main components can effectively improve the renal function of patients with diabetic nephropathy and delay the progress of diabetic nephropathy. However, the results of this study need further research to be confirmed because of the uncertainty of the evidence and the suboptimal risk bias.

## 1. Introduction

Diabetic kidney disease (DKD) is considered as a serious complication of diabetes mellitus (DM) and the main cause of end-stage renal disease (ESRD) [1]. Traditional treatment methods have not achieved satisfactory results in many clinical treatment practices of DKD. It should be noted that TCM has a long history of treating diabetic complications [2], especially diabetic nephropathy. Because of its multitarget effect, Chinese medicine, as the main or alternative treatment for diabetic nephropathy, has good clinical efficacy. In the preclinical research of treating diabetic nephropathy, TCM play a therapeutic role in many ways, such as targeting the inflammatory response, apoptosis, autophagy, and cell stress [3].

*A*stra*galus membranaceus* (AM) is a traditional Chinese medicine from Mongolian milkvetch or Membranaceus milkvetch. It has a longstanding history and has gained widespread clinical applications in China, with the functions of decreasing blood lipid, lowering blood sugar, eliminating edema, and so on [4, 5]. In addition, it has been proven to have wide pharmacological effects on diabetes and its complications, particularly in DN, where it has a unique curative effect [6]. *Panax notoginseng (Burk) F.H. Chen* (Sanqi in Chinese) is a commonly used Chinese medicine. Pharmacological studies have shown that notoginseng and its extracts have many functions, such as antioxidation, anti-inflammatory, regulation of blood pressure and blood glucose, inhibition of platelet aggregation, inhibition of neuronal apoptosis, and neuronal protection [7–11].

At present, both Radix Astragali and Radix notoginseng are widely used in the clinical treatment of diabetic nephropathy, but there is still no rigorous systematic evaluation to evaluate the safety and effectiveness of their clinical use in the treatment of diabetic nephropathy with the combination of the two drugs. Now, systematic evaluation is being conducted to provide some reference for clinical popularization and use.

## 2. Methods

### 2.1. Search Strategy

The databases Cochrane Library, PubMed, Embase, Web of Science, the China National Knowledge Infrastructure (CNKI), Wanfang, VIP, and China Biology Medicine (CBM) were systematically searched from the date of their inception until June 2022. No language restriction was imposed. The following search terms were used: diabetic kidney disease, diabetic nephropathy, and Astragali, *Astragalus*, *Astragalus membranaceus*, *Astragalus* plant, *Astragalus* root, milkvetch root, Radix Astragali, and Sanqi, Sanqizongzaogan, *Panax notoginseng*, *Panax notoginseng* saponins, Lulutong, Xuesaitong, and Xueshuantong. An example search using PubMed is provided in Table 1.

### 2.2. Inclusion and Exclusion Criteria

Studies meeting the following criteria were included: (1) Types of studies: clinical RCTs using ARPN as a treatment for DKD; (2) Types of participants: the diagnostic criterion for DKD included adults with primary diabetes and persistent albuminuria/proteinuria, details are as follows: urinary albumin-to-creatinine ratio(UACR) ≥ 30 mg/g or urinary albumin excretion rate (UAER)≥ 30 mg/24 h and repeatedly check UACR or UAER within 3 to 6 months, and the critical value was reached or exceeded twice in 3 times; (3) Types of interventions and comparisons: RCTs comparing ARPN therapy versus control therapy were included (control therapy were ACEI/ARB or monotherapy including AR or PN or basic treatment.). (4) Outcomes: clinical efficacy, albuminuria, 24 h urinary protein, serum creatinine (Scr), blood urea nitrogen (BUN), glycosylated hemoglobin (HbAlc), total cholesterol (TC), triglycerides (TG), low-density lipoprotein cholesterol (LDL), and TCM symptom score.

The exclusion criteria were: (1) duplicate studies; (2) reviews, animal, or cell experiments; (3) non-RCTs, case reports, meta-analysis, meeting abstracts, and editorials; (4) incomplete, incorrect, or not available studies data, or the study did not report at least one of the primary outcomes; (5) studies, in which control groups received an intervention that treatment groups did not receive; and (6) improper outcome measures.

The primary outcomes were albuminuria and 24 h urinary protein. Secondary outcomes included clinical efficacy (CE), serum creatinine (Scr), blood urea nitrogen (BUN), HbA1c, total cholesterol (TC), triglyceride (TG), low-density lipoprotein (LDL), and TCM symptom score (TSS). Studies that failed to report at least one of these primary outcomes were excluded.

### 2.3. Selection of Studies and Data Extraction

Two reviewers independently conducted the comprehensive search, and a third reviewer checked for consistency. Discrepancies about screening results were resolved by a discussion and reaching consensus. The reviewers deleted duplicate records, screened the relevance of titles and abstracts, and identified those that they were excluded or requiring further evaluation. We then read the full texts to make sure that all the selected articles meet the inclusion criteria. The following data were extracted from each study: first author and study year, interventions, comparisons, number of patients, duration, and results report.

### 2.4. Statistical Analysis

Data analysis was performed by using the Review Manager 5.4.0 and the Stata 15 statistical package. For continuous outcomes, mean and standard mean (SD) were obtained from each study and presented as standard mean difference (SMD) or mean difference (MD). The random effects model was used to calculate the pooled SMD when substantial heterogeneity occurred (*P* < 0.05 or *I*^2^ > 50%). Publication/reporting biases were performed by using funnel plots. Subgroup analyzes were conducted to explore potential sources, where results were stratified by factors such as different control group treatments, including ACEI/ARB, TCM, and basic treatment.

## 3. Results

### 3.1. Study Identification and Selection

A total of 315 records were retrieved in the initial database search. After deleting 23 duplicate articles, 292 records are eligible for further analysis. According to the inclusion and exclusion criteria, 246 articles were excluded after reading the titles and abstracts. The remaining 76 full-text articles were evaluated. Studies with an irrelevant study design, non-RCTs, meta-analysis, and studies that reported only combination treatment were excluded.

**Figure 1:**
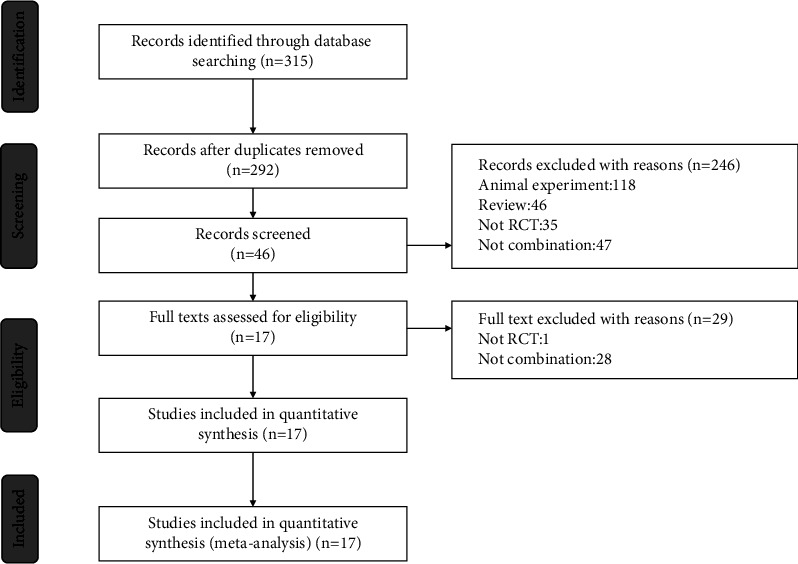
PRISMA flow diagram of screening process.

Finally, a total of 17 RCTs were included in the meta-analysis. The selection process is carried out according to the Preferred Reporting Items for Systematic Reviews and Meta-analyzes (PRISMA) guidelines, as shown in Figure 1 [12].

### 3.2. Study Characteristics

Details of the study characteristics were presented in Table 2. A total of 17 studies involving 1342 patients with diabetic nephropathy were included in this study, which covered the period from 2002 to 2018. Among the 17 RCTs, 9 studies evaluated ARPN plus ACEI/ARB compared with ACEI/ARB; six studies assessed ARPN compared with single *Astragalus membranaceus* or Panax notoginseng, and two studies evaluated ARPN plus basic treatment, including keep on a diet, lower high blood sugar and high blood pressure, etc.

### 3.3. Quality Assessment

**Figure 2:**
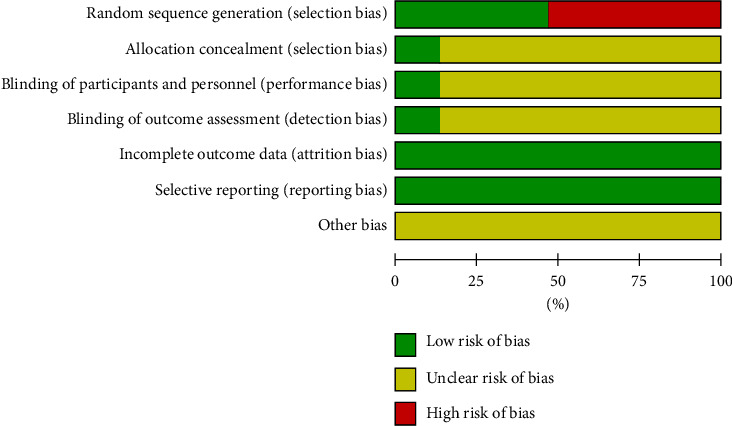
Summary of risk-of-bias assessment of included studies.

The overall quality of the methodology of the included studies was low because of unmasked participants and personnel, unclear randomized and allocated procedures. The risk of bias outcomes is summarized in Figure 2. Eight articles were randomized by a random number table. The remaining studies did not mention the details of randomization. All the included trials did not describe the concealment, blinding, and case-shedding method.

### 3.4. Primary Outcomes

**Figure 3:**
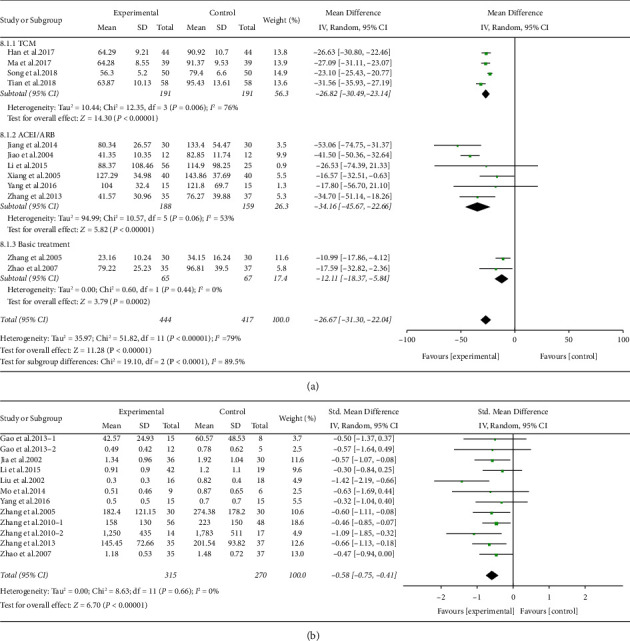
Forest plot of albuminuria (a) and 24 h urinary protein (b) outcome.

Effects of ARPN on albuminuria and 24 h urinary protein Twelve studies with 861 participants reported data on urinary albumin excretion rate at the end of treatment. We classified the studies into three groups based on the treatment of the control group: TCM, ACEI/ARB, and basic treatment. Because of high heterogeneity, the random-effect model was used, as shown in Figure 3(a). Compared with the control group, the UAER level of the treatment group decreased significantly (SMD −26.67, 95% CI −31.30 to −22.04, *P* < 0.00001) with heterogeneity (*I*^2^ = 79%). Subgroup analysis suggested that the treatment of the control group could be the sources of heterogeneity. All of the 3 subgroups notably reduced UAER compared with the control group with lower heterogeneity (TCM group: SMD −26.82, 95% CI −30.49 to −23.14, *P* < 0.00001, *I*^2^ = 76%. ACEI/ARB group: SMD −34.16, 95% CI −45.67 to −22.66, *P* < 0.00001, *I*^2^ = 53%. Basic treatment group: SMD −12.11, 95% CI −18.37 to −5.84, *P* < 0.001, *I*^2^ = 0%).

A total of ten studies with 585 participants compared 24 h urinary protein levels between the treatment and control group. Compared with the control group, the ARPN group apparently reduced the 24 h urinary protein (SMD −0.58, 95% CI −0.75 to −0.41, *P* < 0.00001) without heterogeneity (*I*^2^ = 0%). As shown in Figure 3(b).

### 3.5. Secondary Outcomes

#### 3.5.1. Effect of ARPN on Clinical Efficacy

**Figure 4:**
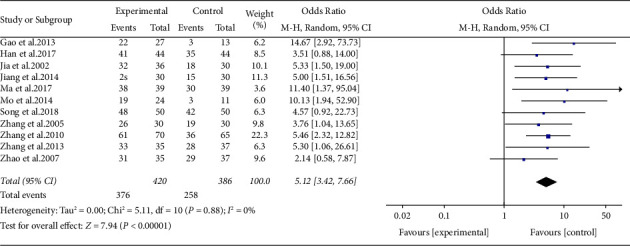
Forest plot of clinical efficacy outcome.

Eleven studies analyzed the total effective rate of ARPN. According to the clinical symptoms and laboratory examination, the curative effect of ARPN can be divided into obvious effective, effective, and invalid, and the total effective rate includes the proportion of obvious effective and effective. Compared with the control group, the total effective rate of the treatment group increased, with statistical significance (SMD 5.12, 95% CI 3.42 to 7.66, *P* < 0.00001) with good homogeneity (*I*^2^ = 0%), as shown in Figure 4.

#### 3.5.2. Effect of ARPN on Kidney Function

**Figure 5:**
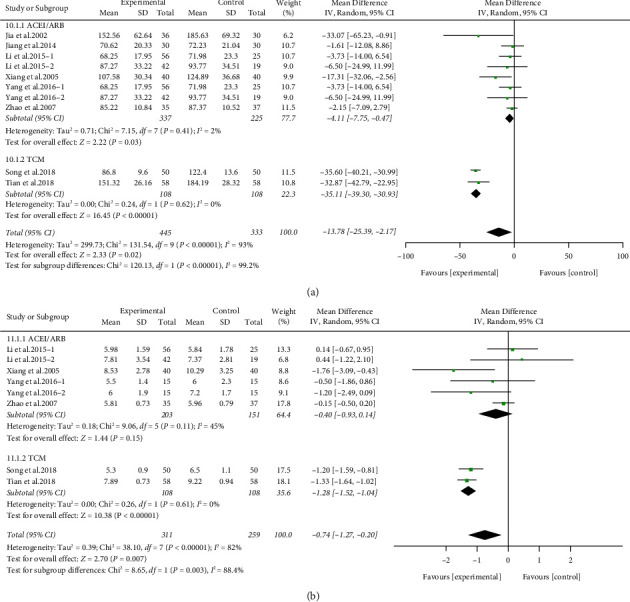
Forest plot of Scr (a) and BUN (b) outcome.

Kidney function was reflected by the measurement of serum creatinine concentration (Scr) and blood urea nitrogen (BUN) in the included studies. Scr levels at the end of treatment were evaluated in 778 participants from eight studies. Studies were classified into an ACEI/ARB group and TCM group based on the treatment of the control group. Scr level of the treatment group decreased significantly (SMD −13.78, 95% CI −25.39 to −2.17, *P* < 0.05) with high heterogeneity (*I*^2^ = 93%) compared with the control group. Subgroup analysis suggested that both of the two subgroups significantly reduced Scr compared with the control group with no heterogeneity (ACEI/ARB group: SMD −4.11, 95% CI −7.75 to −0.47, *P* < 0.05, *I*^2^ = 2%. TCM group: SMD −35.11, 95% CI −39.30 to −30.93, *P* < 0.00001, *I*^2^ = 0%). The treatment of the control group could be the sources of heterogeneity (Figure 5(a)).

The similar result was found in the BUN analysis. BUN levels at the end of treatment were evaluated in 570 participants from six studies. Compared with control group, ARPN group apparently reduced the BUN level (SMD −0.74, 95% CI −1.27 to −0.20, *P* < 0.01) with high heterogeneity (*I*^2^ = 82%). Subgroup analysis shown that there was no significant difference between ACEI/ARB group and control group (SMD −0.40, 95% CI −0.93 to −0.14, *P*=0.15, *I*^2^ = 45%), while the TCM group had statistical significance (SMD −1.28, 95% CI −1.52 to −1.04, *P* < 0.00001, *I*^2^ = 0%). Subgroup analysis revealed that different control group treatments may have been the source of heterogeneity (Figure 5(b)).

#### 3.5.3. Effect of ARPN on HbA1c

**Figure 6:**
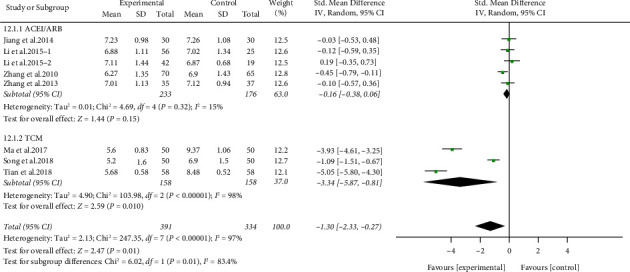
Forest plot of HbA1c outcome.

Seven studies, including 725 participants, mentioned HbA1c. Studies were classified into an ACEI/ARB group and TCM group based on the treatment of the control group. Compared with control group, ARPN group apparently reduced the HbA1c (SMD −1.30,95% CI −2.33 to −0.27, *P* < 0.05) with high heterogeneity (*I*^2^ = 97%). Subgroup analysis suggested that no significant differences in HbA1c reduction were observed in ACEI/ARB group, whereas the TCM group was more effective in HbA1c reduction (ACEI/ARB group: SMD −0.16, 95% CI −0.38 to 0.06, *P*=0.15, *I*^2^ = 15%. TCM group: SMD −3.34, 95% CI −5.87 to −0.81, *P* < 0.05, *I*^2^ = 98%). The heterogeneity among subgroups was large (*I*^2^ = 83.4%) (Figure 6).

#### 3.5.4. Effect of ARPN on Blood Lipid

**Figure 7:**
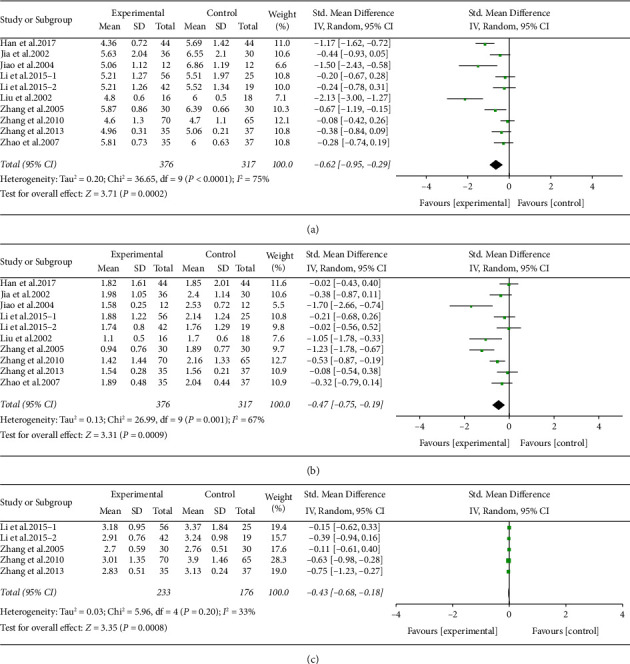
Forest plot of TC (a), TG (b), and LDL (c) outcome.

Nine studies, including 693 participants, mentioned the TC and TG concentrations. Compared with the control group, the ARPN group apparently reduced the TC and TG concentrations level (TC: SMD −0.62, 95% CI −0.95 to −0.29, *P* < 0.001. TG: SMD −0.47, 95% CI −0.75 to −0.19, *P* < 0.001) with high heterogeneity (TC: *I*^2^ = 75%, TG: *I*^2^ = 67%) (Figures 7(a) and 7(b)). Sources of heterogeneity were not identified.

Four studies, including 409 participants, mentioned the LDL-C concentrations. Compared with the control group, the ARPN group apparently reduced the LDL-C concentrations level (SMD −0.43,95% CI −0.68 to −0.18, *P* < 0.001) without heterogeneity (*I*^2^ = 33%) (Figure 7(c)).

#### 3.5.5. Effect of ARPN on TCM Symptom Score

**Figure 8:**
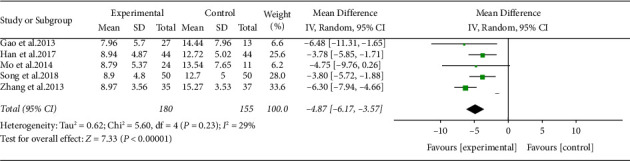
Forest plot of TCM symptom score outcome.

Five studies with 335 participants compared TCM symptom score between the ARPN group and control group. Compared with control group, ARPN group apparently reduced TCM symptom score (MD −4.87, 95% CI −6.17 to −3.57, *P* < 0.00001) without heterogeneity (*I*^2^ = 29%). As shown in Figure 8.

### 3.6. Publication Bias

**Figure 9:**
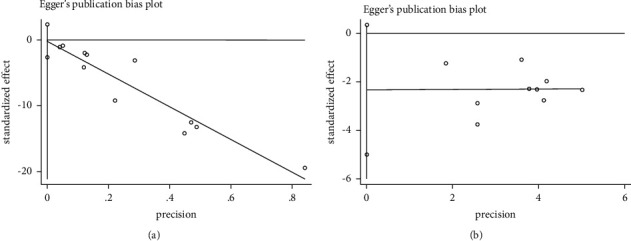
Publication bias of UAER (a) and 24 h proteinuria (b).

Publication bias was not detected in the primary outcomes of UAER (*P* > 0.05, Figure 9(a)) and 24 h proteinuria (*P* > 0.05, Figure 9(b)). The results show that there is no publication offset.

### 3.7. Quality Evaluation of GRADE

Based on the main relevant outcome indicators included in the study, and referring to the GRADE evaluation standard of evidence quality, this study comprehensively evaluates the evidence quality from five aspects: bias risk, evidence consistency, evidence directness, evidence accuracy, and publication bias. When the combined evidence results have one of the above situations, the corresponding evidence quality will be degraded, and the evidence quality of the main outcome indicators in this study will be comprehensively judged based on the aforementioned possible degradation factors. The evaluation results suggest that the evidence quality of the main therapeutic outcomes related to DKD treated by ARPN is “moderate” or “low.” As shown in Table 3.

### 3.8. Safety Evaluation

Safety outcomes were reported in 3 out of 17 included studies [15, 21, 27]. The authors described that the participants had a slight adverse effect, such as dizziness, but the symptoms gradually decreased with the treatment, and there was no statistical difference between the two groups.

## 4. Discussion

Because the pathogenesis of DN is complex, it is difficult to delay the progress of DN effectively by single drug acting on a certain target. Traditional Chinese medicine (TCM) is playing an increasingly important role in the prevention and treatment of DN because of its advantages of multitarget and combined medication. It is believed that the pathogenesis of diabetic nephropathy is qi deficiency and blood stasis, so the method of invigorating qi and activating blood circulation is the fundamental treatment of DN, which should run through the whole treatment of DN [30]. *Astragalus membranaceus* and *Panax notoginseng*, as traditional Chinese medicines, have their main functions of invigorating qi and activating blood, respectively [31, 32]. The combination of them accords with the basic treatment of invigorating qi and activating blood and has a solid theoretical basis.

Toxicological studies show that the combination of *Astragalus membranaceus* and *Panax notoginseng* is safe, and no genetic mutation, target organ damage, or other toxic reactions have been observed in cell and animal experiments [33]. Radix notoginseng Compound (A&P) is a traditional Chinese medicine compound composed of Radix Astragali, Radix notoginseng, Radix *Angelicae sinensis*, Achyranthis Radix, and Thallus laminariae, which is widely used in China to treat CKD. It has been found that A&P combined with Bifidobacterium can downregulate the inflammatory reaction of macrophages in the kidney and intestine by inhibiting a small molecular signaling pathway, thus protecting the kidney from the influence of CKD, which provides a new idea for traditional drug treatment of CKD [34]. In diabetic nephropathy, *Astragalus* mongholicus Bunge and *Panax notoginseng* (Burkill) F.H. Chen formula (APF) significantly improved the renal damage of DN mice, especially recovered blood urea nitrogen, serum creatinine, and 24-hour proteinuria. In STZ-induced diabetic nephropathy mice, APF also decreased the mRNA and protein expression of inflammatory factors such as TNF-*α*, IL-1-*β*, and IL-6, and also improved the damage of high glucose to renal mesangial cells by regulating autophagy [35]. In addition, the combination of *Astragalus membranaceus* and notoginseng Radix can also improve kidney podocyte injury in diabetic rats, and the curative effect is significantly better than that of *Astragalus membranaceus* or notoginseng Radix alone [36]. Many clinical studies have shown that the combination of *Astragalus membranaceus* and *Panax notoginseng* can improve the renal function of diabetic nephropathy patients.

In this study, we aimed to analyze the effect of ARPN on renal function of diabetic nephropathy patients and included 17 clinical studies involving 1342 diabetic nephropathy patients, which involved compatibility of *Astragalus membranaceus* and notoginseng Radix or traditional Chinese medicine compound mainly composed of *Astragalus membranaceus* and notoginseng Radix. The analysis shows that ARPN can significantly improve the clinical effective rate of diabetic nephropathy, and the curative effect of reducing UAER and 24 h UP is also significantly better than that of the control group, and it can also improve the renal function. In addition, it can also reduce glycosylated hemoglobin and blood lipid, and improve the TCM syndrome score. However, subgroup analysis showed that compared with the control group's basic treatment or only using *Astragalus membranaceus* and notoginseng Radix, when the control group's treatment plan was ACEI/ARB drugs, the curative effects of Scr, BUN, and HbA1c in the treatment group and the control group decreased. In the analysis of Scr, the *P* value of ACEI/ARB group is higher than that of TCM group. The analysis of BUN shows that there is no statistical significance between the ACEI/ARB treatment group and control group, but there is significant statistical significance between the TCM treatment group and the control group. The same result also appears in the analysis of HbA1c, which may be due to the renal protective effect of ACEI/ARB drugs. As we all know, ACEI/ARB drugs have been widely used in DKD treatment. Their effectiveness in reducing or preventing microalbuminuria has been documented [8, 9]. ACEI/ARB drugs can reduce proteinuria and improve renal function in patients with DKD. Therefore, with the intervention of ACEI/ARB drugs, the protective effect of ARPN on the kidney cannot be fully exerted.

Overall, the quality of the evidence in this study is moderate or low and the results of the risk bias analysis show that many studies have high or unclear risks. Although all the included studies indicated that a randomized controlled design was carried out, only 8 studies described the randomization method in detail, that is, using computer-generated random numbers or random number tables. Two studies described the blind method in detail, and adopted the standard double-blind method. Other studies lacked the description of blind method. None of the included studies mentioned distribution concealment. In addition, some test indexes are highly heterogeneous. After using randomized model or subgroup analysis, it is found that the treatment plan of the control group is the source of heterogeneity of some indexes. In addition, due to the limitation of language barrier, this study only searched Chinese and English databases, and all the included studies were conducted in China, which affected the final results to some extent. Therefore, the applicability of the results in other countries needs further study.

## Figures and Tables

**Table 1:** Search strategy for PubMed.

Search strategy	Search terms
*Astragalus membranaceus* (#1)	((((((Astragali) OR (*Astragalus*)) OR (*Astragalus membranaceus*)) OR (*Astragalus* plant)) OR (*Astragalus* root)) OR (milkvetch root)) OR (Radix Astragali) OR (Huangqi)
*Panax notoginseng* (#2)	(((((((*Panax notoginseng*) OR (*Panax notoginseng* saponins)) OR (Radix notoginseng)) OR (Radix *Panax notoginseng*)) OR (Sanqi)) OR (Lulutong)) OR (Xuesaitong)) OR (Xueshuantong)
Diabetic kidney disease (#3)	((((diabetes nephropathy) OR (diabetic nephropathy)) OR (diabetic nephrosis)) OR (renal diabetes)) OR (diabetic kidney disease)
#4 ((#1) AND (#2) AND (#3))	((((((((Astragali) OR (*Astragalus)*) OR (*Astragalus membranaceus*)) OR (*Astragalus* plant)) OR (*Astragalus* root)) OR (milkvetch root)) OR (Radix Astragali) OR (Huangqi)) AND ((((((((*Panax notoginseng*) OR (*Panax notoginseng* saponins)) OR (Radix notoginseng)) OR (Radix *Panax notoginseng*)) OR (Sanqi)) OR (Lulutong)) OR (Xuesaitong)) OR (Xueshuantong))) AND (((((diabetes nephropathy) OR (diabetic nephropathy)) OR (diabetic nephrosis)) OR (renal diabetes)) OR (diabetic kidney disease))

**Table 2:** Characteristics of included studies.

Study years	Treatment	Control	Number (T/C)	Duration	Results report
Zhang et al. 2010 [13]	Qishen Yiqi droplet + Telmisartan	Telmisartan	69/66	3 months	CE, 24UP, HbA1c, TC, TG, LDL
Han et al. 2017 [14]	Shenqi Jiangtang granule + Xueshuantong capsule	Xueshuantong capsule	44/44	2 months	CE, UAER, TC, TG, TSS
Song et al. 2018 [15]	Shenqi Jiangtang granule + Xueshuantong capsule	Shenqi Jiangtang granule	50/50	2 months	CE, UAER, Scr, BUN, HbA1c, TSS
Jiao et al. 2004 [16]	*Astragalus* + Xuesaitong injection + Irbesartan	Irbesartan	12/12	1 months	UAER, TC, TG
Liu et al. 2002 [17]	*Astragalus* + Xuesaitong injection + Benazepril	Benazepril	24/26	3 weeks	24UP, TC, TG
Jia et al. 2002 [18]	*Astragalus* + Xueshuantong injection + Captopril	Captopril	39/27	1 months	CE, 24UP, Scr, TC, TG
Ma et al. 2017 [19]	Shenqi Jiangtang granule + Xueshuantong capsule	Xueshuantong capsule	39/39	2 months	CE, UAER, HbA1c
Tian et al. 2018 [20]	Shenqi Jiangtang granule + Xueshuantong capsule	Xueshuantong capsule	58/58	2 months	UAER, Scr, BUN, HbA1c
Mo et al. 2014 [21]	Tangshen formula	Tangshen simulated granule	26/13	3 months	CE, 24UP, TSS
Zhao et al. 2007 [22]	Tangqing granules + basic treatment	Basic treatment	40/40	1 months	CE, UAER, 24UP, Scr, BUN, TC, TG
Zhang et al. 2005 [23]	*Astragalus* + Xuesaitong injection + basic treatment	Basic treatment	30/30	3 weeks	CE, UAER, 24UP, TC, TG, LDL
Xiang et al. 2005 [24]	Tangshen granules + Irbesartan	Irbesartan	40/40	2 months	UAER, Scr, BUN
Zhang et al. 2013 [25]	Shenqi Jiangtang granule + Xueshuantong capsule + ACEI/ARB	ACEI/ARB	35/37	2 months	CE, UAER, 24UP, HbA1c, TC, TG, LDL, TSS
Yang et al. 2016 [26]	Tangshen Formula + ACEI/ARB	ACEI/ARB	122/58	6 months	UAER, 24UP, Scr, BUN
Li et al. 2015 [27]	Tangshen Formula + ACEI/ARB	ACEI/ARB	117/44	6 months	24UP, Scr, BUN, HbA1c, TC, TG, LDL
Jiang et al. 2014 [28]	Qishen Yiqi droplet + Telmisartan	Telmisartan	30/30	3 months	CE, UAER, Scr, HbA1c
Gao et al. 2013 [29]	Treatment granule	Simulated granule	27/13	3 months	CE, 24UP, TSS

**Table 3:** Quality evaluation of GRADE.

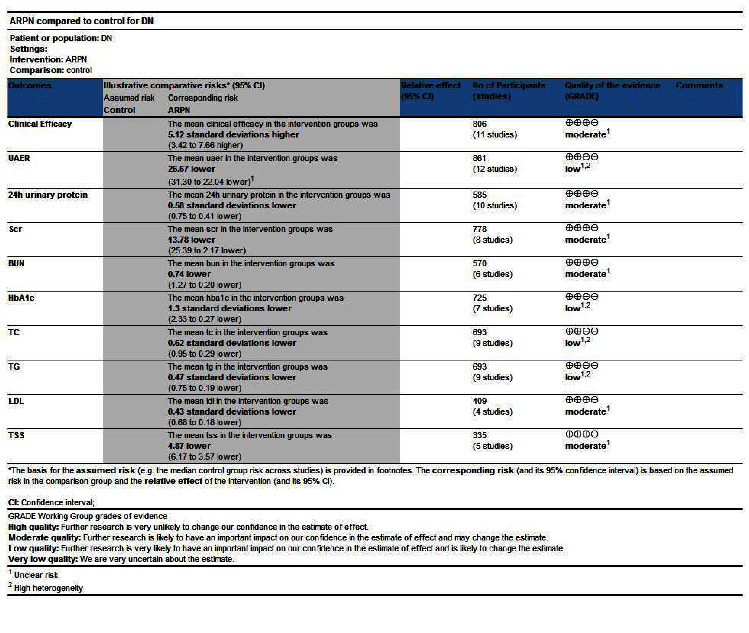

## Data Availability

All data that support the findings of this study are available from the corresponding author upon reasonable request.
